# The inter-connections between self-harm and aggressive behaviours: A general network analysis study of dual harm

**DOI:** 10.3389/fpsyt.2022.953764

**Published:** 2022-07-22

**Authors:** Matina Shafti, Sarah Steeg, Derek de Beurs, Daniel Pratt, Andrew Forrester, Roger T. Webb, Peter James Taylor

**Affiliations:** ^1^Manchester Academic Health Science Centre, The University of Manchester, Manchester, United Kingdom; ^2^Division of Psychology and Mental Health, Centre for Mental Health and Safety, School of Health Sciences, University of Manchester, Manchester, United Kingdom; ^3^Centre for New Treatments and Understanding in Mental Health (CeNTrUM), University of Manchester, Manchester, United Kingdom; ^4^Trimbos Institute (Netherlands Institute of Mental Health and Addiction), Utrecht, Netherlands; ^5^Division of Psychological Medicine and Clinical Neurosciences, School of Medicine, Cardiff University, Cardiff, United Kingdom; ^6^National Institute for Health and Care Research (NIHR) Greater Manchester Patient Safety Translational Research Centre, University of Manchester and Northern Care Alliance NHS Foundation Trust, Manchester, United Kingdom

**Keywords:** dual harm, self-harm, aggression, co-occurrence, violence, ALSPAC

## Abstract

Dual harm is the co-occurrence of self-harm and aggression during an individual’s lifetime. This behaviour is especially prevalent within criminal justice and forensic settings. The forms of aggression that should be included in the definition of dual harm have not yet been established. This study aimed to use network analysis to inform an evidence-based definition of dual harm by assessing the relationship between self-harm and different forms of aggressive behaviour in young people (*N* = 3,579). We used data from the Avon Longitudinal Study of Parents and Children (ALSPAC). Results revealed low correlations between the variables, leading to sparse network models with weak connections. We found that when separated into their distinct forms, aggressive acts and self-harm are only weakly correlated. Our work provides preliminary evidence to assist in understanding and managing dual harm within clinical and forensic settings and informs recommendations for future research.

## Introduction

Rather than engage in self-harm *or* aggression (i.e., sole harm), some individuals will show both behaviours during their lifetime; this is referred to as dual harm (1). Up to 5% of individuals living in the community have been reported to engage in dual harm (2–5). This figure rises to 11–15 and 19–56% in prisons and forensic mental health services, respectively, indicating that dual harm is of particular concern amongst forensic and criminal justice populations (6–10). Self-harm and aggression have been reported to increase and peak during adolescence, underlining the importance of interventions that target harmful behaviours during this period (11–13). Richmond-Rakerd et al.’s (5) study of adolescents found that twins who had engaged in self-harm were three times more likely to perpetrate a violent crime compared to their co-twins who had not engaged in self-harm. By examining differences between twins raised in the same family, these findings highlight an association between self-harm and aggression amongst young people, in which self-harm is a predictor of aggression risk independent of genetic or familial factors.

There is evidence that, compared to persons with a history of sole harm, individuals who have engaged in dual harm are more likely to have had various harmful experiences during adolescence, including adverse events (e.g., maltreatment, family violence, neglect), psychotic symptoms, substance dependence, and traits relating to interpersonal and emotional problems (5, 6, 14–16). Therefore, early intervention models that target risk factors during adolescence may be effective in preventing the development of dual harm. The importance of early prevention is demonstrated by findings showing that individuals who engage in dual harm show a riskier pattern of behaviours and are more likely to experience negative outcomes, including higher risk of dying from external causes (5, 9, 17). Negative outcomes have especially been highlighted within forensic and criminal justice settings. Despite forming a minority, it has been reported that prisoners with a history of dual harm spend 40% longer in prison and twice as much time in segregation compared to those who engage in aggression alone (1). These findings highlight the limited effectiveness of current strategies in helping those who engage in dual harm, as well as the importance of preventing this behaviour before it arises in forensic and criminal justice settings (18). It is important that we investigate dual harm during adolescence to thereby learn how this behaviour may emerge and develop.

Despite the duality of self-harm and aggression in a subset of affected individuals, research and practice tend to make a separation between these two behaviours. Consequently, we have limited knowledge of the understanding and management of dual harm within clinical and forensic services. There is no agreed definition of dual harm, making it challenging to reach an evidence-based conclusion regarding the nature, determinants and consequences of this behaviour. Whilst dual harm includes both self-harm and aggression, it is unclear which exact harmful behaviours should be included under these broad terms. Self-harm is a broad term that encompasses both suicidal and non-suicidal forms of self-directed harm, covering a range of behaviours, including physical self-injury (e.g., self-cutting) and overdose. Aggression may range in severity from minor behaviours (e.g., verbal aggression) to more extreme behaviours (e.g., physical violence), and only a minority of aggressive or violent episodes result in arrests, criminal charges or convictions. Whilst some studies assess self-harm and physical violence when examining dual harm (e.g., 4), others expand their definition by also assessing behaviours such as property damage and verbal aggression (19). Studies tend to measure dual harm by cross-tabulating responses to separate questionnaires of self-harm and aggression. This method has led to inconsistency of measurements and conceptualisations of dual harm, leading to difficulties with comparisons in the literature.

To strengthen our understanding of dual harm, it is important that we first arrive at an empirically derived definition of this behaviour. Within the context of dual harm, self-harm and aggression are thought to be linked (10, 18, 20). Therefore, one way of informing an agreed definition of dual harm is to assess how various aggressive behaviours are associated with self-harm and with each other. For example, incorporating aggressive acts that are strongly associated with self-harm and with each other could lead to a more clinically meaningful definition of dual harm. Whilst there is evidence that self-harm and aggression are correlated, it is less clear which forms of aggressive acts contribute to this association (20). Studies tend to assess aggression more generally by combining items that measure different forms of aggression into one construct. Consequently, it is unclear as to which aggressive behaviours are relevant to consider when assessing dual harm.

Therefore, our study aimed to delineate between separate aggressive acts and assess how these behaviours and self-harm could be interrelated amongst young people within a network model. By investigating how harmful behaviours are associated with each other during their key stage of development in adolescence, findings may inform an evidence-based definition of dual harm that suggests how this behaviour should be understood and measured within research and practice.

## Methods

We used data from the Avon Longitudinal Study of Parents and Children (ALSPAC) – a longitudinal population-based birth cohort study (21–23). We chose this dataset, as variables relevant to our research question were available (Appendix 1). ALSPAC researchers collected data from children born to pregnancies and their parents between April 1991 and December 1992 at regular intervals since birth. The initial number of pregnancies enrolled in the study was 14,541. When the oldest children within this sample were approximately 7 years old, there was further recruitment of children from the initial cohort who had not initially joined the study. ALSPAC is a three-generation study and the present work used data from the G1 generation. This generation is the original cohort, in which there are 68 data collection time-points from birth to 18 years old. The protocol for this study was pre-registered in the Open Science Framework.^1^

We assessed the following variables for the purpose of this study:

### Self-harm

Data about self-harm were obtained through a self-completed questionnaire when participants were, on average, 16.5 years of age. Participants were asked if they had ever “hurt themselves on purpose in any way.” Those who answered “yes” were then asked the frequency at which they had self-harmed in the past year.

### Physical aggression, verbal aggression, property damage, arson, and violence toward animals

Participants self-reported the frequency to which they had engaged in the above aggressive behaviours over the past year. Participants were, on average, 15.5 years of age when they reported these behaviours. Items included “hit/kicked/punched someone,” “threatened to hurt someone,” “rowdy or rude in a public place,” “deliberately damaged or destroyed property,” “set fire or tried to set fire to something,” and “hurt or injured animals or birds on purpose.”

### Bullying

This behaviour was assessed when participants were, on average, 12.5 years, using the Bullying and Friendship Interview Schedule (24). Participants were asked whether they had perpetrated various aspects of bullying, including “threatened/blackmailed,” “hit/beaten up,” and “called someone nasty names.”

### Dating violence

This behaviour was assessed when participants were, on average, 13.5 years, using an interview that consisted of items obtained from a revised version of the Conflict Tactics Scale (25). The interviewer asked participants whether they had intentionally used any of the seven behaviours in the context of dating or romantic relationships. Behaviours included “scratched,” “slapped,” “kicked,” “bent fingers,” “pushed/grabbed/shoved,” “thrown something,” “hit with their fist,” or “another form of violence.”

The study website contains details of all data items that are available in ALSPAC through a fully searchable data dictionary and variable search tool.^2^ The items can be accessed by searching for specific codes (see Supplementary Material) in the variable search tool.

Ethical approval for the study was obtained from the ALSPAC Ethics and Law Committee and the Local Research Ethics Committees.^3^ Informed consent for the use of data collected *via* questionnaires and clinics was obtained from participants following the recommendations of the ALSPAC Ethics and Law Committee at the time.

## Analysis

Missing data were removed using listwise deletion. Network analysis (26) was then applied to assess how self-harm and various aggressive behaviours were connected to each other. Four models were computed using the Mixed Graphical Model approach within R’s mgm package (version 3.6.3) (26–28). We fitted four extra models as the addition of each new variable led to a decrease in sample size due to missing data. Therefore, we aimed to assess whether the addition of variables and changes to sample size would affect the associations between the harmful behaviours. All models were estimated using the “bootnet” package and visualised with the “qgraph” package (29, 30). In each model, variables were represented by nodes that connected to each other *via* edges. Participants who had complete data for all variables in each model were included in the analysis. The first model consisted of self-harm, physical aggression, verbal aggression, property damage, arson, and violence toward animals, comprising 3,579 individuals. For the second model, bullying was added, comprising 3,366 individuals. For the third model, instead of bullying, dating violence was included, comprising 2,043 individuals. Finally, the fourth model consisted of all the above variables, and comprised of 1,981 individuals.

We also examined the following *post hoc* question: what is the association between self-harm and aggression when all aggressive behaviours are considered together as one construct? To answer this, we calculated the correlation between self-harm and all aggressive behaviours by creating one composite aggression variable. This was done by summing the items for the separate aggressive variables into one composite variable.

Given differences in frequency at which self-harm and violence occur between males and females (31–33), we also examined the following *post hoc* question: how does the relationship between harmful behaviours differ between males and females? This was done by computing two gender-specific network models that assessed the interconnections between all harmful behaviours in males and females separately.

Furthermore, we calculated the prevalence of dual harm and sole harm amongst the 1,981 individuals who had complete data for all examined variables. Given the varied conceptualisations of dual harm across the literature, to allow comparability between previously reported studies, we only considered physical violence when calculating prevalence rates. This is because physical violence is typically included in all conceptualisations of dual harm. Therefore, we examined the prevalence of dual harm by identifying those who had engaged in both self-harm and physical violence.

## Results

Five percent of individuals had engaged in both self-harm and physical violence (i.e., dual harm, *n* = 105), 14% had engaged in self-harm alone (*n* = 269) and 16% had engaged in physical violence alone (*n* = 319).

**FIGURE 1:**
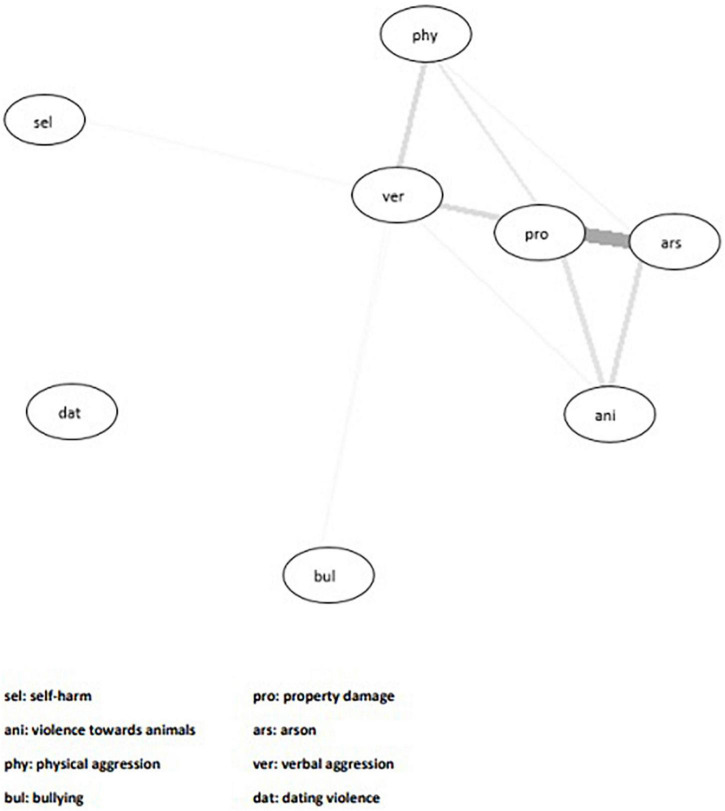
Network model showing the interconnections between all harmful behaviour variables in the entire sample. A connection between two variables is demonstrated by an edge between nodes.

The computed models did not show strong connections between nodes, resulting in sparse networks with mostly weak edges or no evident edge (Appendix 2). Figure 1 shows the network model with all the variables of interest. The weak networks should be attributed to the low bivariate correlations between the variables, with 18 correlations estimated at *r* < 0.20. Specifically, all the correlations between self-harm and the different aggressive behaviours were small, ranging from *r* = −0.03 to 0.12 (Table 1). In contrast, there was more variability between the distinct forms of aggression, with correlations ranging from *r* = 0.02 to 0.48. Table 2 presents the adjacency matrix between all variables of interest in the network model. The adjacency matrix represents partial correlations, where the association between two variables is the association that is left when controlling for all other variables within the network model. Where there was an edge present between nodes (i.e., two variables were connected in the model), this is indicated by 1, whereas 0 indicates that there was no edge between the two nodes.

**TABLE 1:** Correlation matrix between all harmful behaviours examined in the study.

	Property damage	Violence toward animals	Arson	Physical aggression	Verbal aggression	Bullying	Dating violence
Self-harm	0.06*	−0.03	0.09**	0.04	0.12**	0.08**	0.1**
Property damage		0.24**	0.48**	0.35**	0.47**	0.11**	0.05*
Violence toward animals			0.25**	0.13**	0.2**	0.06*	0.02
Arson				0.28**	0.39**	0.09**	0.03
Physical aggression					0.47**	0.15**	0.08**
Verbal aggression						0.16**	0.11**
Bullying							0.08**

*p < 0.05, **p < 0.001.

**TABLE 2:** Adjacency matrix for the network model examining interconnections between all the harmful behaviour variables.

	Self-harm	Physical aggression	Verbal aggression	Property damage	Violence toward animals	Arson	Bullying	Dating violence
Self-harm	–	0	1	0	0	0	0	0
Physical aggression	–	–	1	1	0	1	0	0
Verbal aggression	–	–	–	1	1	0	1	0
Property damage	–	–	–	–	1	1	0	0
Violence toward animals	–	–	–	–	–	1	0	0
Arson	–	–	–	–	–	–	0	0
Bullying	–	–	–	–	–	–	–	0

Given the low correlation between self-harm and the separate aggressive behaviours, we examined how self-harm is associated with aggression when all aggressive behaviours are considered as one construct. The analysis revealed a correlation of *r* = 0.15. Whilst this represents a weak relationship, the correlation coefficient was higher than those found between self-harm and each individual aggressive behaviour.

We also carried out gender-specific analyses examining whether there are differences in how harmful behaviours are related to each other between males and females. The computed network models and adjacency matrix are shown in Supplementary Material (Appendix 3). The network model for males consisted of 826 individuals, and for females, 1,153 individuals. Three percent (*n* = 28) of males engaged in dual harm, compared to 7% (*n* = 77) of females. In the network model for males, no edge was present between self-harm and any of the aggressive behaviours. Nevertheless, the aggressive behaviours in this model were grouped together and shown to be linked by the presence of multiple edges connecting different harmful behaviours to each other. In contrast, the network model for females showed that aggressive behaviours were not as interconnected. However, there was an edge present between self-harm and arson, indicating that these two behaviours are linked.

## Discussion

Findings from this study revealed weak correlations between different forms of aggression and self-harm, resulting in network models with weak connections between nodes. Whilst there is evidence that self-harm and aggression are associated with each other (20), it may be that when aggression is distinguished into its specific forms, this association becomes less apparent. This may be demonstrated by findings that when aggressive behaviours were combined into one variable, the albeit weak correlation between aggression and self-harm was somewhat stronger when compared to the very weak associations between self-harm and most of the separate aggressive variables. A higher correlation would be expected when variables are combined. This may highlight that the correlation found between self-harm and aggression in previous research may be inflated as a result of not distinguishing between distinct forms of aggressive behaviours.

The network models show that whilst the bullying and dating violence nodes are further apart, other aggressive behaviours, such as verbal aggression, property damage and arson, tend to cluster together. This suggests that relational forms of aggression may arise from distinct processes compared to non-relational aggressive behaviours and should not be included in our definition of dual harm. These findings highlight the potential importance of delineating different types of aggression when conceptualising dual harm, as it may be more clinically meaningful to consider aggressive behaviours that have stronger associations with each other and self-harm.

*Post hoc* analysis revealed a higher prevalence of dual harm in females, as well as differences between males and females in how harmful behaviours are connected to each other. It should be noted that these network models may be unstable given the small number of dual harm cases that are present in each, and should therefore be interpreted with caution. Nevertheless, findings may suggest that the aetiologies of dual harm and of harmful behaviours differ somewhat between the genders. Whilst we found no connection between self-harm and aggressive behaviours in males, self-harm may be connected to arson in females. Previous research has found that self-harm and arson have the same psychological processes in women (e.g., communicating distress), suggesting that there may be a shared causal pathway that underlies these behaviours (34). There are differences in the reported prevalence rates of harmful behaviours between males and females. Whilst higher rates of self-harm have been reported in females (31), research has suggested that the prevalence rates of aggression in males and females differ based on the form of violent behaviour being examined (32, 33). Such findings highlight the importance of assessing gender-specific differences in co-occurring violence and self-harm, as well as determining which aggressive behaviours to include in defining dual harm.

The weak connections in our network analysis may be attributed to the study sample. To the best of our knowledge, this is the first study to examine the association between self-harm and different aggressive behaviours in young people within a network model. There is evidence that the pattern and aetiology of harmful behaviours differs across age, suggesting developmental differences in self-harm and aggression (35–37). As such, it may be that the nature of harmful behaviours amongst adolescents is distinct to that of adults and an aetiological link between self-harm and aggression is less apparent amongst younger populations. Furthermore, harmful behaviours, including dual harm, have been shown to be more prevalent amongst clinical and forensic populations than among persons living in the community (2–10, 15). Therefore, it may be that the associations between harmful behaviours are stronger in high-risk populations, such as those in forensic settings.

It should be noted that the prevalence of dual harm in other studies of adolescents living in the community has been reported to be between 4.7 and 31.1% (5, 15, 38, 39). This distinction in prevalence rates may reflect differences in methodology, including definitions of harmful behaviours. For example, Gould et al.’s (38) study, which reported a higher dual harm prevalence of 31.1% adopted a broad definition of aggression by assessing a wide range of items in their measure, including torturing animals, bullying, losing your temper, and arguing with adults at school. On the other hand, Richmond-Rakerd et al. (5) only assessed violent crime when examining aggression, which may have accounted for the lower dual harm prevalence of 4.7%. Given the range of reported prevalence rates, it is challenging to determine the degree our sample is representative of the wider population. These studies highlight the importance of establishing an agreed definition of dual harm to facilitate comparability across all studies reported in the literature.

Although the network models demonstrated weak connections, our findings nevertheless revealed the presence of dual harm amongst an adolescent sample and associations between various harmful behaviours that are present early on in life. Such findings may have implications for clinical management at the level of both services and the individual. For services, given that persons who engage in dual harm are more likely to be in contact with criminal justice and health services, it may be important to adopt more robust coordinated and integrated approaches within these sectors that recognise the relationship between self-harm and aggression. At the individual level, this relationship should be considered and built into assessment, management and intervention processes to enable effective prevention and to reduce the co-occurrence of self-harm and aggression within clinical and forensic settings. Furthermore, research of adolescents and prisoner samples has revealed that those who engage in dual harm are more likely to use more severe self-harm methods compared to those who engage in self-harm alone (5, 9). Therefore, early and systematic consideration of the duality of harmful behaviours may not only help reduce the likelihood of aggression in those who have self-harmed and vice versa, but also reduce lethal risk to self among those who engage in dual harm.

The limitations of our study ought to be considered. Harmful behaviours were assessed at different time points and so age may have confounded the observed results. As with aggression, self-harm is a broad term that includes non-suicidal self-injury (self-harm without intent to die) and suicidal behaviour (self-harm with intent to end one’s life). Our study assessed self-harm more broadly by not distinguishing between these behaviours. Future research should aim to assess differences in how non-suicidal self-injury and suicidal behaviours may be associated with aggression in those who have engaged in dual harm. Furthermore, most harmful behaviours were assessed over a 1 year period. A longitudinal study in which harmful behaviours are measured over a longer time period may reveal stronger associations. The data used in this study were collected *via* self-report. However, there is evidence that both self-harm and aggression are underreported, which may have contributed to the lack of strong correlations between the variables in this study (40, 41). Future investigations should assess the relationship between different aggressive behaviours and self-harm using more than one data source to generate more accurate findings (e.g., self-report, informant-report, official administrative databases). Finally, given that dual harm is especially prevalent within forensic settings, future research should examine the link between self-harm and aggressive behaviours amongst forensic and criminal justice populations.

In conclusion, this study found weak connections between self-harm and specific types of aggressive behaviour amongst adolescents. Nevertheless, the network models highlighted associations between harmful behaviours during adolescence and provide preliminary evidence that relational forms of aggression should not be included in an established definition of dual harm. By following our recommendations for future research, studies may be able to provide more robust findings as regards to how dual harm should be conceptualised within both academic research and clinical practice. Identifying an evidence-based conceptualisation of dual harm will help inform the development of more effective management strategies aiming to address dual harm within forensic and clinical settings.

## Data availability statement

The raw data supporting the conclusions of this article will be made available by the authors, without undue reservation.

## Ethics statement

Ethical approval for the study was obtained from the ALSPAC Ethics and Law Committee and the Local Research Ethics Committees (http://www.bristol.ac.uk/alspac/researchers/research-ethics/). Informed consent for the use of data collected *via* questionnaires and clinics was obtained from participants following the recommendations of the ALSPAC Ethics and Law Committee at the time. Written informed consent to participate in this study was provided by the participants’ legal guardian/next of kin.

## Author contributions

MS was responsible for the data analysis and wrote a first draft of the manuscript, with all authors contributing to the final manuscript. All authors contributed to the formulation of research questions and design of the study.

## Conflict of interest

The authors declare that the research was conducted in the absence of any commercial or financial relationships that could be construed as a potential conflict of interest.

## Publisher’s note

All claims expressed in this article are solely those of the authors and do not necessarily represent those of their affiliated organizations, or those of the publisher, the editors and the reviewers. Any product that may be evaluated in this article, or claim that may be made by its manufacturer, is not guaranteed or endorsed by the publisher.

## Footnotes

1
https://osf.io/fpcgb

2
http://www.bristol.ac.uk/alspac/researchers/our-data/

3
http://www.bristol.ac.uk/alspac/researchers/research-ethics/
