# Supplementary material for: The inter-connections between self-harm and aggressive behaviours: A general network analysis study of dual harm
Source: Front Psychiatry. 2022 Jul 22;13:953764. doi: 10.3389/fpsyt.2022.953764 (PMC9354883; doi:10.3389/fpsyt.2022.953764)
Supplement: Supplementary file 1 [file Data_Sheet_1.docx]

**Supplementary Material**

**Appendix 1. Variables in this study and codes**

| **Variable** | **Description** | **Age measured** | **Source of measurement** | **Code** |
| --- | --- | --- | --- | --- |
| Physical aggression | Frequency that they have kicked/punched/hit their sibling or someone else in past year | 15.5 | Child-completed questionnaire | fh8313 |
| Verbal aggression | Frequency that they have threatened to hurt someone they know in the last year or been rowdy or rude in a public place such that people complained or got them in trouble in the last year | 15.5 | Child-completed questionnaire | fh8308, fh8321 |
| Property damage | Frequency that they have deliberately damaged or destroyed property in the last year | 15.5 | Child-completed questionnaire | fh8314 |
| Arson | Frequency that they have set fire or tried to set fire to something on purpose in the last year | 15.5 | Child-completed questionnaire | fh8319 |
| Violence towards animals | Frequency that they have hurt or injured animals on purpose in the last year | 15.5 | Child-completed questionnaire | fh8318 |
| Self-harm | Number of times they have purposely hurt themselves in past year | 16.5 | Child-completed questionnaire | ccs6530, ccs6531 |
| Bullying | Frequency they have threatened/blackmailed a peer, hit/beaten up a peer, or called a peer nasty names | 12.5 | Child-completed questionnaire | ff6110, ff6120, ff6140 |
| Dating violence | Frequency they have engaged in various forms of dating violence | 13.5 | Child-completed questionnaire | fg4320, fg4322, fg4324, fg4326, fg4328, fg4330, fg4332, fg4335 |

**Appendix 2. Network models**

Key -

sel: self-harm pro: property damage

ani: violence towards animals ars: arson

phy: physical aggression ver: verbal aggression

bul: bullying dat: dating violence

Model 1: Network model showing the interconnections between the primary harmful behaviour variables in the entire sample.


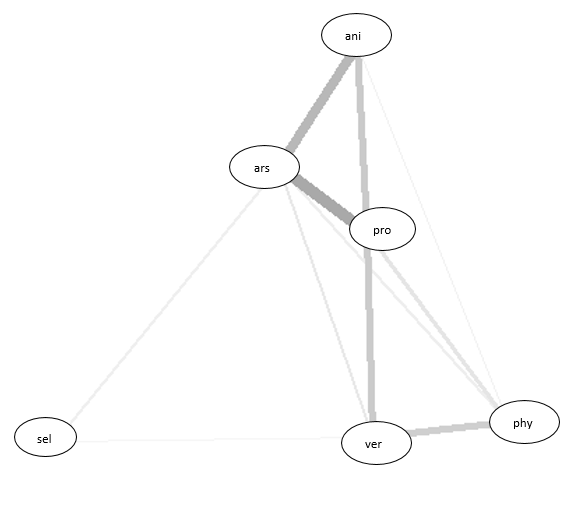


Model 2: Network model showing the interconnections between the primary harmful behaviour variables, as well as bullying, in the entire sample.


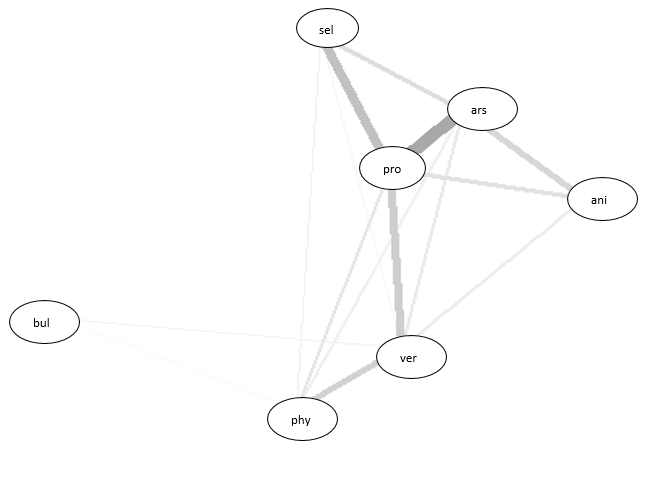


Model 3: Network model showing the interconnections between the primary harmful behaviour variables, as well asdating violence, in the entire sample.


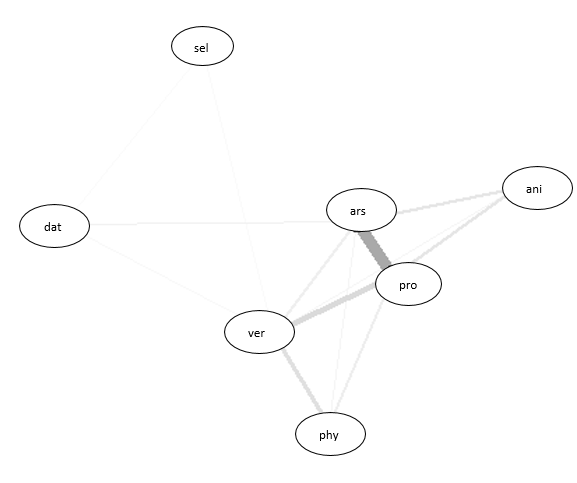


**Appendix 3.** **Gender-specific analyses**

Post-hoc network model showing interconnections between all harmful behaviours in females


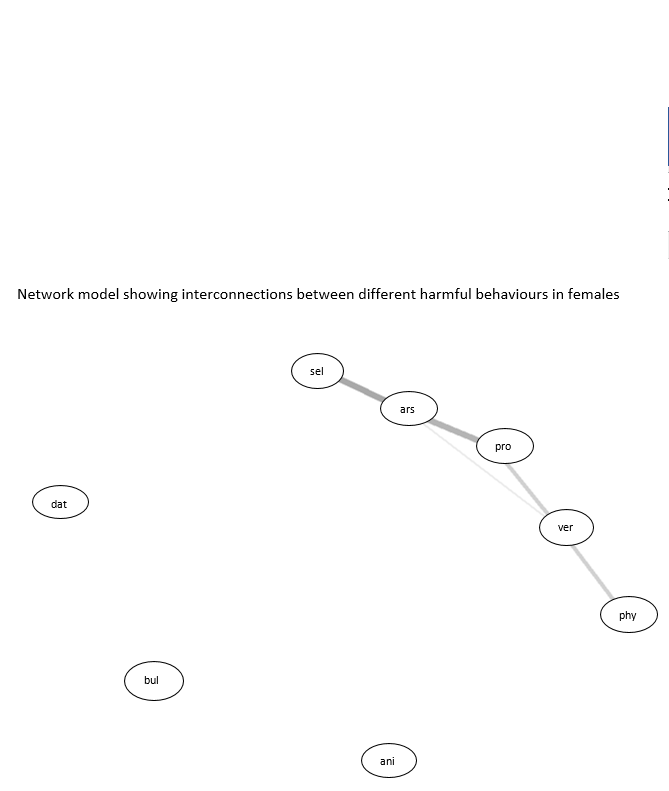


Post-hoc network model showing interconnections between all harmful behaviours in males


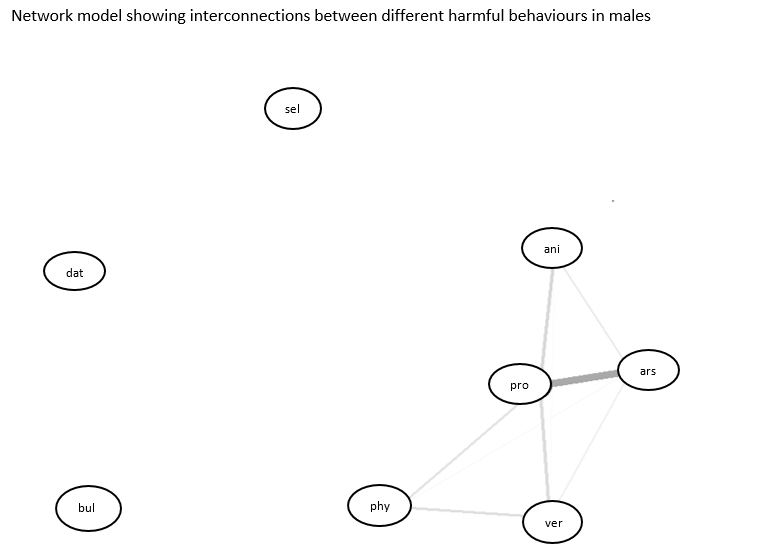


**Table 3.**

*Adjacency matrix for the network model examining interconnections between all the harmful behaviour variables in males*

|  | Self-harm | Physical aggression | Verbal aggression | Property damage | Violence towards animals | Arson | Bullying | Dating violence |
| --- | --- | --- | --- | --- | --- | --- | --- | --- |
| Self-harm | - | 0 | 0 | 0 | 0 | 0 | 0 | 0 |
| Physical aggression | - | - | 1 | 1 | 0 | 1 | 0 | 0 |
| Verbal aggression | - | - | - | 1 | 1 | 1 | 0 | 0 |
| Property damage | - | - | - | - | 0 | 1 | 0 | 0 |
| Violence towards animals | - | - | - | - | - | 1 | 0 | 0 |
| Arson | - | - | - | - | - | - | 0 | 0 |
| Bullying | - | - | - | - | - | - | - | 0 |

**Table 4.**

*Adjacency matrix for the network model examining interconnections between all the harmful behaviour variables in females*

|  | Self-harm | Physical aggression | Verbal aggression | Property damage | Violence towards animals | Arson | Bullying | Dating violence |
| --- | --- | --- | --- | --- | --- | --- | --- | --- |
| Self-harm | - | 0 | 0 | 0 | 0 | 1 | 0 | 0 |
| Physical aggression | - | - | 1 | 0 | 0 | 0 | 0 | 0 |
| Verbal aggression | - | - | - | 1 | 0 | 1 | 0 | 0 |
| Property damage | - | - | - | - | 0 | 1 | 0 | 0 |
| Violence towards animals | - | - | - | - | - | 0 | 0 | 0 |
| Arson | - | - | - | - | - | - | 0 | 0 |
| Bullying | - | - | - | - | - | - | - | 0 |
